# Providing specimen transport through an online marketplace in the Northern region of Ghana

**DOI:** 10.4102/ajlm.v12i1.2062

**Published:** 2023-07-20

**Authors:** Abass Abdul-Karim, David Opare, Ulysses Balis, Lee F. Schroeder

**Affiliations:** 1 Tamale Zonal Public Laboratory, Ghana Health Service, Tamale, Ghana; 2 National Public Health Laboratory, Ghana Health Service, Accra, Ghana; 3 Department of Pathology, University of Michigan School of Medicine, Ann Arbor, Michigan, United States

**Keywords:** laboratory network, specimen transport, specimen referral, public health laboratory, essential diagnostics, diagnostic network

## Abstract

**Background:**

Integrated diagnostic networks, which are themselves dependent on robust specimen transport solutions, are fundamental to effective healthcare systems.

**Objective:**

This study aimed to pilot an online marketplace for the transport of specimens throughout a laboratory network in Ghana.

**Methods:**

Independent drivers were matched with health facilities that required specimen transport using a suite of mobile applications and web portals developed for this study. This marketplace was piloted with seven drivers, two laboratories, and five health facilities in Ghana’s Northern region from March 2019 to October 2019.

**Results:**

During the pilot, 182 deliveries were completed for 691 patients, including 4118 laboratory tests for antenatal care, disease surveillance, and clinical testing. Testing included 34 tests for communicable and non-communicable diseases. All but two specimens (laboratory cancellations) were successfully delivered and tested. The median time from request to encrypted emailing of results was 19.7 h, while that for a drop-off request was 0.9 h. In the midwife registry, the median time from patient visit to result recording was 1 day, compared to 4 days in the same months in 2018, and the number of mothers without documented testing decreased from 41 to 3. Similarly, the proportion of tuberculosis specimen deliveries from Buipe Polyclinic to Tamale Zonal Laboratory taking over 1 day fell from 62% at baseline to 3% during the pilot.

**Conclusion:**

An online marketplace successfully orchestrated the delivery of laboratory specimens under a variety of clinical circumstances, reducing overall turn-around time without diminution of the overall specimen delivery process.

**What this study adds:**

This study established the efficacy of an online marketplace to orchestrate timely and high-quality delivery of specimens within a laboratory network.

## Introduction

Fundamental to any effective national healthcare system is a functional laboratory network with hierarchical tiers.^1,2^ Through a laboratory network, peripheral health centres without full testing capacity can access a larger portfolio of diagnostics by sending patient specimens to higher-tier laboratories. A robust courier solution that ensures specimens are shipped efficiently and reliably is essential to the laboratory network but is often non-existent in many low- and middle-income countries, except for some select diseases of public health importance.^3,4^ In Ghana, specimen transport is limited but critical for clinical testing and disease surveillance and is a particular concern in the northernmost regions of the country.^5,6^

The Maputo Declaration of 2008 called for the strengthening of laboratory networks throughout sub-Saharan Africa.^1^ This was in response to the escalating efforts to curb endemic conditions like HIV, tuberculosis, and malaria. Since then, other major global efforts for the surveillance of antimicrobial resistance and epidemic-prone diseases^2^ like coronavirus disease 2019 have identified the need for tiered laboratory networks.^7,8,9^ Tiered laboratory networks permit knowledge sharing between tiers, strengthened reagent supply chains, shared resources for instrument servicing, and testing access for remote health facilities through specimen transport.^10^

Recent efforts to strengthen specimen transportation revealed the feasibility of a wide variety of strategies, with perhaps the greatest barrier being a lack of engagement.^3,11,12,13,14,15,16,17^ In Ghana, various ad-hoc approaches to specimen transport are utilised and the results are not always satisfactory. For instance, the time to deliver specimens for meningitis testing, a disease requiring rapid results, varied between 3 and 7 days in the Yendi and Talensi districts in Ghana.^5,18^ The rate of laboratory-confirmed meningitis is thus low in Ghana and throughout the entire meningitis belt, where fewer than 10% of cases are confirmed through testing.^19^

Online marketplaces like Uber™ and Airbnb™ have had major impacts in multiple industries. Uber Technologies Incorporated (San Francisco, California, United States) revolutionised the transportation industry by tapping into the sharing economy. This study aimed to create and pilot an online marketplace in Ghana to match drivers with health facilities that need specimens sent to higher-tier laboratories. Our goal was to harness the existing transportation resources in Ghana to efficiently move specimens throughout the laboratory network. This approach also allowed for the most effective use of diagnostic resources in a health system where instruments are rarely used to full capacity due to a lack of locally available specimens.

## Methods

### Ethical considerations

This study was conducted according to the ethical approval provided by Ghana Health Service Ethical Review Committee (GHS-ERC 002/05/18). Written consent was obtained for participation from the prospective survey participants, as well as from patients submitting samples to be used in the study. All data were anonymised and maintained on *Health Insurance Portability and Accountability Act*-compliant servers and password-protected laptops.

### Development of the mobile and web-based application

**FIGURE 1:**
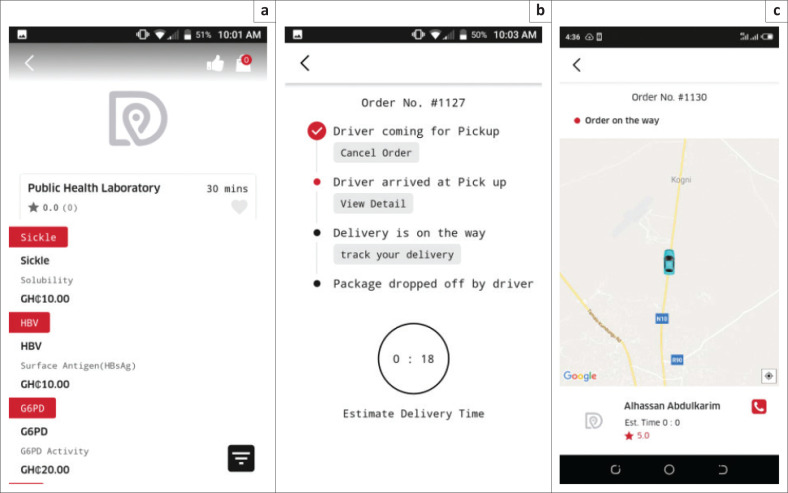
Screenshots of the health facility mobile phone application developed for the specimen transport network piloted at health facilities in the Northern region of Ghana between March 2019 and October 2019. The application includes (a) the laboratory test menus from which healthcare staff select tests, (b) the tracking features of the application, and (c) visualisation of real-time driver location during the delivery.

A mobile and web-based application suite formed the technological foundation for our online marketplace (Figure 1). The custom suite was created for this study by App Emporio (Rajkot, India) on a technological stack including NodeJS (https://nodejs.org/en), Angular2 (https://angular.io/), and MongoDB (https://www.mongodb.com/). The suite included different persona-based applications for health facilities, drivers, and laboratories. There was also a web portal for the participating laboratories, where test menus, turn-around times (TATs), and prices could be added. Similarly, an administrative portal provided real-time visualisation of operations, including global positioning system locations of drivers and active deliveries.

### Workflow of the specimen transport network

Patients were identified through routine clinical care and brought to the health facility’s laboratory for phlebotomy and possible participation in the pilot. There, specimens were collected from the patients and packaged in triple containers with ice packs and paper requisitions to facilitate proper identification of samples and confirmation of tests to be performed.^20^ The packages were then sealed to prevent exposure of drivers to potential biohazards. Drivers were thus not required to use personal protective equipment. A trained health facility liaison then used the application to request a driver from a list of active drivers, sorted by distance. If the desired driver did not accept, the application automatically queried other drivers in order of distance from the pick-up point, based upon real-time geospatial telemetry from the driver-persona-based application. Once a driver accepted the task, they travelled to the health facility, picked up the package, and delivered the same to the selected laboratory. During this time, the driver was trackable in real time through the application or web interface. Upon delivery, the driver confirmed completion through the application, and the laboratory confirmed receipt of the adequate samples for testing, as well as the availability of the required testing reagents. Laboratory test results were delivered to the health facilities electronically through Proton AG (Geneva, Switzerland), a secure end-to-end encrypted email service. Quality assurance staff from the project team monitored the requested deliveries and ensured that samples arrived safely and that the laboratories performed the tests and communicated the results. The application also included a rating system for health facilities and drivers.

A pilot study of the online marketplace was conducted from March 2019 to October 2019 at five public health facilities in the Northern region of Ghana (Online Supplementary Document – Supplementary Methods), and a medical records review collected data on test utilisation and TATs (Online Supplementary Document – Supplementary Methods). Additionally, prospective surveys of patients (Online Supplementary Document – Supplementary Survey 1) and providers (Online Supplementary Document – Supplementary Survey 2) collected data on challenges and preferences for laboratory testing.

### Data analysis

All data on specimen delivery were stored on our servers and made available through our web administration dashboard. No private health information was stored on this server. Data from the abstraction of medical records and laboratory registers were entered into Microsoft Excel spreadsheets (Microsoft Corporation, Redmond, Washington, United States) and checked for consistency. Data were imported to the R statistical environment (v3.6.1)^21^ for statistical analysis. For dichotomous outcomes, statistical evaluations were conducted using the McNemar test (mcnemar.test function within the R *stats* package) with continuity correction, and TATs were compared using the two-sample Kolmogorov-Smirnov test (ks.test function within the R *stats* package), with approximated *p*-values where exact computation was not possible due to ties (*p* < 0.05 were considered significant).

Due to technical difficulties, deliveries were often cancelled and had to be re-requested by facilities after the driver had already started the collection or delivery. In these instances, timestamps would underestimate actual delivery times. Therefore, in the TAT calculations, any deliveries with collection or delivery durations of less than 10 min were excluded, as the shortest driving time between the health facilities and laboratories was about 10 min.

## Results

In the medical record review of 15 health facilities (pre-pilot), 23 laboratories were identified that performed external testing, with a median of four laboratories per health facility (Online Supplementary Document – Supplementary Figure 1). Outside Tamale, test results for 99% of in-house tests were delivered by the laboratory within 1 day of request, compared to 82% of tests sent to external laboratories (*p* < 0.001). In Tamale, the proportions of test results resulted by the laboratories within 1 day of test request were similar for in-house tests (100%) and tests conducted at other laboratories (98%). For health facilities outside Tamale, test results for 90% of in-house tests were recorded by providers within the same day of the test request compared to 74% of tests performed outside the facility (*p* < 0.001). Within Tamale, test results for 51% of in-house tests were recorded on the same day of the test request compared to 40% of tests performed outside the facility (*p* = 0.50). Within Tamale, the median TAT from test request to recording of results was 3.1 h for in-house testing compared to 1.7 h for send-out testing (*p* < 0.001). Likewise, the median TAT from request to recording by the provider was higher for in-house testing (12.7 h) compared to send-out testing (4.8 h; *p* = 0.08). The types of tests conducted in-house were similar to those conducted elsewhere, with a total of 23 different tests identified. The most common of these were malaria, complete blood count, haemoglobin, typhoid, urinalysis with microscopy, sickle solubility, urine pregnancy, and hepatitis B surface antigen tests, which collectively accounted for 95% of testing. Other less-commonly ordered tests included stool microscopy, hepatitis C test, glucose-6-phosphate dehydrogenase test, tuberculosis sputum microscopy, liver function tests, renal function tests, glucose tests, and HIV tests.

**FIGURE 2:**
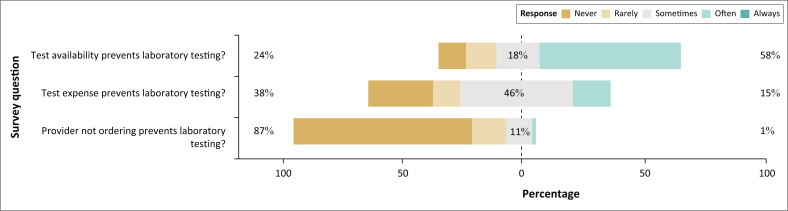
Results of a patient survey conducted at health facilities in the Northern region of Ghana between March 2019 and October 2019. Patients reported that test availability was the most common cause preventing laboratory testing.

Interviews were conducted with 73 patients, including representatives from each of our pilot sites. When asked about the frequency with which they experienced different barriers to testing, 58% of patients described test availability as ‘often’ or ‘always’ a barrier, 15% reported test expense as ‘often’ or ‘always’ a barrier, and 1% reported the physician not ordering tests as ‘often’ or ‘always’ a barrier (Figure 2). There were no responses of ‘Other’ reasons. Nearly equal numbers of patients responded that they would either transport the samples to a laboratory themselves (44%) or that a specimen would be sent by some other means (46%). Others (8%) reported that they had experienced both, while 1% reported that they had experienced neither. Due to the structure of the question, it was difficult to interpret how patient samples were transported to outside laboratories (e.g., by a system organised by the health facility or by the patient’s family or friends). Nearly all patients (96%) said they would be interested in the specimen transport service being piloted, mostly to avoid travel or reduce transport costs; two patients were, however, concerned about the possibility of mislabelled specimens or loss of confidentiality.

**FIGURE 3:**
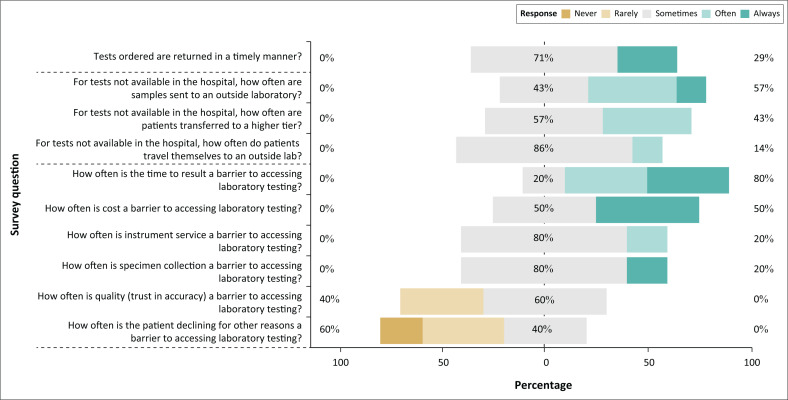
Results of a survey conducted among health providers at health facilities in the Northern region of Ghana between March 2019 and October 2019. Related questions are grouped between dashed lines.

Seven providers were interviewed, with representation from each health facility (Figure 3). Twenty-nine percent responded that tests are ‘often’ or ‘always’ returned promptly. When testing was not available in their health facility, 57% of providers responded that samples were ‘often’ or ‘always’ sent to outside laboratories, 43% stated that patients were transferred to higher-tier facilities, and 14% stated that patients travelled themselves to outside laboratories. The most commonly experienced barriers to accessing diagnostic testing were TAT (80% responded ‘often’ or ‘always’) and cost (50% responded ‘often’ or ‘always’). All seven providers said that the specimen transport services would be useful if made available at their facilities.

Over the 8 months of the pilot, 182 deliveries were conducted for 691 patients, including 4118 laboratory tests (Table 1). Other than laboratory cancellations for two specimens due to spillage (samples were re-collected), all deliveries were completed, and results were posted. Of the 182 deliveries, 120 were excluded from TAT calculations as their collection or delivery durations were less than 10 min, indicating a technical difficulty with the application that required the resubmission of delivery requests. At the individual testing level, the median time from health facility request to sending of results by encrypted email was 19.7 h, with an interquartile range (IQR) of 18.6 h – 20.7 h. The median time from request to collection of the specimen by the driver was 21 min (IQR: 11 min – 38 min), and the median time from specimen pick-up to drop-off at the laboratory was 28.8 min (IQR: 19 min – 65 min). The median time from drop-off to reporting of results was 18.5 h (IQR: 16.2 h – 19.4 h), accounting for 94% of the total TAT (IQR: 89% – 96%). Turn-around times observed during the pilot could not be compared with those observed during the retrospective analysis because most medical records only contained dates, not times (hours and minutes). Driver fees varied from $1.75 United States dollars (USD) to $8.77 USD per delivery, depending on the requesting facility. Multiple patients were pooled for each delivery. Some surveillance testing for tuberculosis required variably higher fees for delivery due to longer travel times (> 2 h; up to $17.54 USD). The direct payments to drivers on average were $0.77 USD per patient and $0.11 USD per test, excluding the longer travel to the Buipe District for surveillance testing (18 deliveries).

**TABLE 1:** Characteristics and performance of the specimen transport network piloted at health facilities in the Northern region of Ghana between March 2019 and October 2019.

Network characteristics	Value
**Network structure**	
Total number of health facilities	5
Total number of drivers	7
Total number of laboratories	2
**Network performance**	
Total number of deliveries	182
Total number of patients	691
Total number of tests	4118
**Turn-around times (median)**	
Request to collect†	0.4 h
Collect to deliver‡	0.5 h
Deliver to laboratory result§	18.5 h
Time to send results to facility¶	0.3 h
**Fees paid to drivers per delivery (United States dollars)**	
Bilpela (3 km)	$1.75
Zuo (10 km)	$3.51
Savelugu (27 km)	$8.77
Kings Medical Centre (31 km)	$8.77
Buipe District for surveillance (variable km)	≤ $17.54

† , Request to collect: time from health facility request for pickup within the application to the time that the package was picked up by the delivery person.

‡ , Collect to deliver: time that the package was picked up by the delivery person to the time the package was delivered to the laboratory.

§ , Delivery to laboratory result: time the package was delivered to the laboratory to the time the laboratory produced a result.

¶ , Time to send results to facility: time the result was produced in the laboratory to the time results were sent by email to the requesting health facility.

**FIGURE 4:**
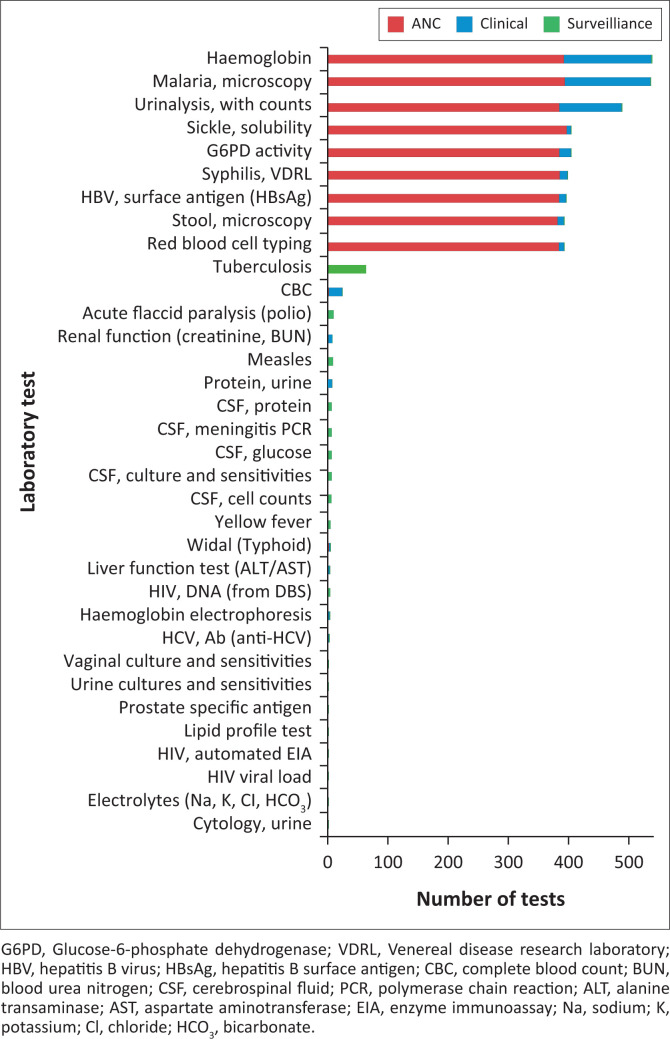
Laboratory tests ordered during the pilot of a specimen transport network at health facilities in the Northern region of Ghana between March 2019 and October 2019. The figure shows tests performed for antenatal care, general clinical care, and public health surveillance. Antenatal care guidelines recommend a specific set of tests, which explains the consistent ordering patterns for that patient population. However, much general clinical testing included the same tests as for antenatal care. Surveillance testing was largely for tuberculosis but also included testing for bacterial meningitis and other priority public health targets.

One hundred and ten deliveries (60% of all deliveries) included samples for clinical testing (Figure 4). Deliveries were conducted mostly for haemoglobin (*n* = 146 tests), malaria (*n* = 142), and urinalysis (*n* = 103) testing. Surveillance testing initiated by disease control officers was included in 18% of deliveries, and these included tests for tuberculosis (*n* = 63), meningitis (*n* = 5), measles (*n* = 7), and yellow fever (*n* = 4).

**FIGURE 5:**
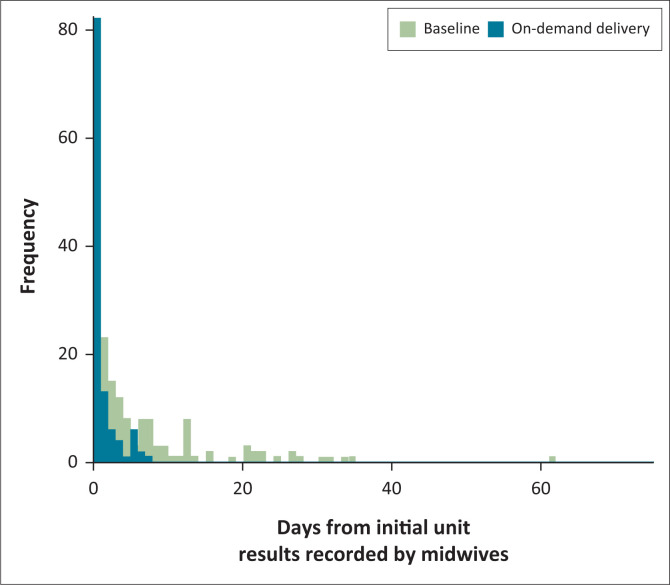
Antenatal care testing times and screening rates as extracted from a midwife registry in the Northern region of Ghana between May 2018 – August 2018 (baseline) and May 2019 – August 2019 (on-demand delivery). The histograms represent the number of patients (*y*-axis) with documented test results within a given time frame from the initial patient visit (*x*-axis). The bars are overlaid on top of each other.

At the antenatal care (ANC) facility reviewed, there were 248 mothers at baseline and 250 in the pilot phase. More mothers were without any documented testing in the ANC facility during the baseline months in 2018 (41 not tested; 207 tested) compared to the corresponding months during the pilot (3 not tested; 247 tested; McNemar test: *p* < 0.001). In the baseline months, 136 mothers had dates recorded in the midwife register for when their laboratory test results were entered. During the pilot, laboratory test result dates were entered in the register for 115 mothers. The median time from the initial visit to result recording in the ANC register (which may be later than the actual return of results) was significantly shorter during the pilot compared to the baseline months (1 day vs 4 days; two-sample Kolmogorov-Smirnov test: *p* < 0.001) (Figure 5).

**FIGURE 6:**
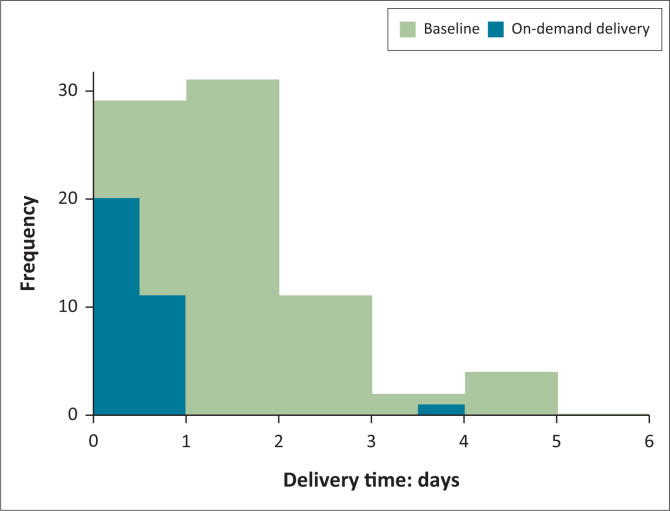
Specimen transport times for tuberculosis testing as documented in the Public Health Laboratory register from the Buipe polyclinic in the Northern region of Ghana between 2009 and 2014 (baseline) and March 2019 – October 2019 (on-demand delivery). The histogram shows the number of specimens (*y*-axis) and the time intervals within which they were delivered for testing after specimen pick-up (*x*-axis). The bars are overlaid on top of each other.

In the review of zonal laboratory records for tuberculosis testing, there were 77 specimens during the baseline months and 32 specimens during the pilot. During the pilot, the median delivery time for specimens was 0 days (same day) compared to a median of 2 days at baseline. This translated to 97% of specimens being delivered in less than 1 day (time from specimen collection to receipt in laboratory) during the pilot compared to 38% of specimens being delivered in less than 1 day at baseline (two-sample Kolmogorov-Smirnov test: *p* < 0.001) (Figure 6).

## Discussion

This pilot of an online marketplace for the transport of laboratory specimens revealed that specimens could be successfully delivered under a variety of clinical circumstances, including for general clinical testing at health facilities and hospitals, ANC testing with daily deliveries of bundled patient specimens, and surveillance testing as requested by disease control officers. For tuberculosis testing, TATs improved from a baseline period, and for ANC testing, both TAT and the number of mothers screened improved.

A 2021 study found that most health facilities in the Northern region of Ghana did not offer the majority of tests recommended in the World Health Organization Essential Diagnostics List.^6^ Our review of medical records revealed that the testing that does occur is performed by a combination of on-site and external laboratories. Outside of testing for HIV early infant diagnosis and some limited diseases for public health surveillance, none of the facilities reviewed operated formalised specimen transport systems for clinical testing (A.A.-K., 2021, personal communication, March 31). Presumably, therefore, samples tested at external laboratories were mostly transported through ad-hoc means (e.g., patient presentation to the laboratory, delivery of the specimen by family or friends, etc.). More on-site tests were completed within 1 day of request compared to external testing, and this difference was greater outside Tamale. This suggests that specimen transport should be prioritised in rural areas, where there are longer distances to external laboratories. Nevertheless, patients in both urban and rural settings would likely benefit from such services.

The sustainable development goals demand quality essential healthcare services with universal health coverage, where timeliness is a key component of quality.^22,23,24,25^ In line with this, the benefits of integrated laboratory networks to healthcare delivery include increased availability, accuracy, and TAT of laboratory testing.^26,27^ During the pilot, the time from the initial visit to recording of test results in the midwife register at the ANC decreased from 4 days to 1 day, and tuberculosis testing times decreased from 2 to 0 days (same day). As specimen transport systems are a key component of laboratory networks, multiple models have been implemented in different settings, and although the published models are not directly comparable (different metrics and different geographic and disease characteristics), they provide some context to the findings in this study. In a 2013 study in Uganda, a motorcycle hub-and-spoke delivery system for centralised HIV early infant diagnosis led to a reduction in TAT from 1–2 months to 5–10 days.^11^ The same service led to a TAT reduction for tuberculosis testing from 21 to 3 days.^3^ In Ethiopia, a private–public partnership involving the Ethiopian Postal Service Enterprise led to HIV early infant diagnosis TATs decreasing from 7 to 2 days in Addis Ababa and from 10 to 5 days in the Amhara region.^3,11,12^ The current pilot performed comparably, if not better than these.

In addition to TAT, laboratory networks can also improve the rates at which patients receive testing and downstream interventions. For example, in a 2015 study in Haiti, the number of patients enrolled on antiretroviral therapy for new sites joining the specimen referral network increased by 182% within 6 months.^28^ Antenatal care screening rates for mothers increased in this study compared to the prior year (3 out of 250 mothers not tested compared to 41 out of 248 mothers not tested at baseline). Antenatal care screening is important for the prevention of mother-to-child transmission of infectious diseases, monitoring for disorders such as pre-eclampsia, and ensuring that expectant mothers with anaemia deliver at facilities with transfusion capabilities. While this study did not follow such patient outcomes, it is reasonable to expect that there would be a commensurate positive impact with improved screening.^29,30^ Regarding tuberculosis, at least six previously undiagnosed patients were diagnosed during the pilot. One of these patients reported prior symptoms for 1 year, and another described her son’s recent death from a condition with similar symptoms. The early detection of tuberculosis, enabled through laboratory networks, can have an important impact on transmission (and thus prevalence), patient outcomes, and the economic burden on affected families.^31,32^

This pilot was designed to assess the feasibility and effectiveness of the transport model, not the costs. Nonetheless, when a health system is determining whether to provide testing on site or via specimen transport, specimen transport cost is a factor that must be considered. In some settings, overall testing costs may be lower with external testing. In the 2013 study in Uganda, the implementation of the specimen transport system for centralised testing led to a 62% reduction in operational costs.^3^ In a 2023 study in Uganda, researchers found similar per-test costs in the decentralised versus centralised model for tuberculosis testing.^33^ Although we documented driver fees, which varied based on distance travelled, we did not perform a comprehensive cost analysis. These deliveries often included batches of patient specimens, thus reducing the per-specimen transportation cost considerably. In this study, excluding the longer deliveries for surveillance in Buipe District, average driver fees were $0.73 USD per patient and $0.11 USD per test. In other settings, this amount would vary based on the use of taxis versus motorcycles (with motorcycles likely costing less), the number of patients per delivery, and the number of tests per delivery. Another cost was for smartphones, as most of the drivers had phones, but not smartphones. The study required providing them with smartphones and data. However, this augmented provisioning to drivers may not be necessary if smartphone adoption increases over time.^34^ A full analysis would need to include the costs of application development and maintenance, platform operation, as well as the economies of scale that depend on the number of deliveries per period, among other costs.

Ghana was the first sub-Saharan African country to introduce a tax-funded National Health Insurance Scheme (NHIS 2003). The NHIS provides payments to accredited public and private laboratories for testing. However, NHIS reimbursement to laboratories is often less than what laboratories would charge when patients pay ‘out of pocket’. A 2021 study in Ghana found that the greater the difference between the NHIS reimbursement and the market rate, the less available the test was in health facilities and laboratories.^6^ Nonetheless, the NHIS represents a foundation upon which payments for diagnostic testing can be provided. The NHIS provides a single payment per test and does not pay for specimen transport, the cost of which would thus be borne by the health facility, the patient (who might otherwise have to pay for transport themselves), or the laboratories, as they desire more specimens. Because such a transport system would increase testing demand, laboratories may be willing to pay for some, or possibly all, of the transport costs. This would be particularly true if specimens are transported in batches such that per-test delivery costs are low compared to testing costs. Regarding sustainability, future work would be needed to further explore the scenarios (e.g., frequency, distance, and urgency of pick-ups) in which this service would be optimal for both the ‘buyer’ and ‘seller’. This includes understanding the ‘willingness to pay’ of the potential buyer. It is also important to understand the ‘willingness to sell’ of the potential seller of this service under each scenario (i.e., drivers). Payment could be improved through telebanking, and different payment contracts could be explored (e.g., simple payment per delivery or flat rates plus per-delivery fee).

### Limitations

There were challenges experienced during this pilot. First, facilities often had to resend requests and that may have impacted TAT estimates, although this was mitigated by excluding unreasonably short collection or delivery durations of less than 10 min. These difficulties could have been due to unstable cellular networks leading to the cancellation of active deliveries. In the next phase, cellular message event buffering with a closed-loop confirmation messaging protocol could mitigate this shortcoming. Second, while the surveillance testing pilot was successful, disease control officers sometimes made requests from very distant locations (> 2-h drive), thus leading to unsustainably high driver fees. Recruitment of bus drivers in a daisy-chained fashion to allow deliveries to and from bus stations and subsequent bus delivery between cities is a possible solution. Third, requests from district hospitals were fewer than anticipated. This may have represented a reluctance to engage in a change of procedure. It is also possible that providers preferred to refer patients to nearby private laboratories where the providers had existing relationships. Further investigation would be necessary to determine the root causes of low district hospital order rates.

### Conclusion

One path towards improving diagnostic testing is additional in-house testing, but a complementary approach using specimen transport could build on the laboratory network approach. Instead of investing in fleets of drivers for this purpose, an online marketplace taps into existing transportation resources that are often under-utilised. Through such a specimen transport service as piloted in this study, existing laboratory capacity could be better harnessed for patient care and disease surveillance.
